# 758 Correlation between initial and diagnostic bronchoalveolar lavage isolates among patients with inhalation injury and pneumonia

**DOI:** 10.1093/jbcr/irad045.233

**Published:** 2023-05-15

**Authors:** Devin Gaskins, Taylor Coston, Saman Arbabi, John McClellan, T Eoin West, Barclay Stewart, Austin Bailey

**Affiliations:** University of Washington School of Medicine, Seattle, Washington; University of Washington, Seattle, Washington; University of Washington and Harborview Medical Center, Seattle, Washington; Harborview Medical Center, Tacoma, Washington; University of Washington,Seattle, Washington; Harborview Medical Center, Rochester, New York; University of Washington School of Medicine, Seattle, Washington; University of Washington, Seattle, Washington; University of Washington and Harborview Medical Center, Seattle, Washington; Harborview Medical Center, Tacoma, Washington; University of Washington,Seattle, Washington; Harborview Medical Center, Rochester, New York; University of Washington School of Medicine, Seattle, Washington; University of Washington, Seattle, Washington; University of Washington and Harborview Medical Center, Seattle, Washington; Harborview Medical Center, Tacoma, Washington; University of Washington,Seattle, Washington; Harborview Medical Center, Rochester, New York; University of Washington School of Medicine, Seattle, Washington; University of Washington, Seattle, Washington; University of Washington and Harborview Medical Center, Seattle, Washington; Harborview Medical Center, Tacoma, Washington; University of Washington,Seattle, Washington; Harborview Medical Center, Rochester, New York; University of Washington School of Medicine, Seattle, Washington; University of Washington, Seattle, Washington; University of Washington and Harborview Medical Center, Seattle, Washington; Harborview Medical Center, Tacoma, Washington; University of Washington,Seattle, Washington; Harborview Medical Center, Rochester, New York; University of Washington School of Medicine, Seattle, Washington; University of Washington, Seattle, Washington; University of Washington and Harborview Medical Center, Seattle, Washington; Harborview Medical Center, Tacoma, Washington; University of Washington,Seattle, Washington; Harborview Medical Center, Rochester, New York

## Abstract

**Introduction:**

Inhalation injury is associated with increased risk of pneumonia. Bronchoscopy with bronchoalveolar lavage (BAL) is commonly used to diagnose and grade inhalation injury while obtaining a microbiological sample. It has been shown that a quarter of patients with inhalation injury have a single bacteria isolated from initial BAL within 12-48 hours of injury. The clinical significance of this and role of antibiotics in this setting are unknown. We investigated the incidence that bacteria isolated during a pneumonia episode were previously isolated on surveillance BAL among patients with inhalation injury.

**Methods:**

Among patients with inhalation injury admitted to a large ABA-verified burn center from 2009-2022, microbiological isolates from initial BAL and subsequent culture obtained during a pneumonia episode (BAL, sputum), were extracted from medical records. Abbreviated injury severity score (AIS) was used to grade inhalation injury. Hospital-acquired pneumonia (HAP) was defined using CDC PNU1 criteria, and ventilator-associated pneumonia (VAP) was defined using PNU2 criteria with BAL cutoff >10,000 colony forming units (CFU).

**Results:**

Two-hundred forty-eight patients with inhalation injury who underwent bronchoscopy for airway inspection and surveillance BAL within 48 hours of injury were included in the analysis. Of the 111 who had a bacterial isolate from initial BAL, 23 (21%) subsequently developed HAP or VAP during the hospitalization. Median (IQR) days between bronchoscopy and pneumonia diagnosis was six (2-8.5). In three of the 11 VAP cases (27%), the organisms identified at pneumonia diagnosis matched the organisms identified on initial BAL. Antibiotic use on admission was not associated with re-culture of initial BAL organism during a subsequent pneumonia episode (p=0.071).

**Conclusions:**

Among patients with inhalation injury and bacteria isolated on surveillance BAL, 21% developed pneumonia during their hospital stay, usually with different bacteria than those isolated at initial bronchoscopy. There was no association between empiric antibiotic use and incidence of initial BAL organisms re-cultured during subsequent pneumonia episode.

**Applicability of Research to Practice:**

Findings suggest initial BAL isolates cannot guide empiric coverage of pneumonia for patients with inhalation injury. This remains an important area of future research.

